# Detection of dynamic lung hyperinflation using cardiopulmonary exercise testing and respiratory function in patients with stable cardiac disease: a multicenter, cross-sectional study

**DOI:** 10.1186/s13102-024-00871-z

**Published:** 2024-04-15

**Authors:** Kazuyuki Kominami, Kazuki Noda, Nanaho Minagawa, Kazuya Yonezawa, Masanori Ueda, Yasuyuki Kobayashi, Makoto Murata, Masatoshi Akino

**Affiliations:** 1 Department of Rehabilitation, Sanseikai Kitano Hospital, 6-30, 1-chome, Kitano 1-jyo, Kiyota-ku, 004-0861 Sapporo, Hokkaido Japan; 2 Department of Rehabilitation, National Hospital Organization Hakodate Hospital, Hakodate, Japan; 3 Sinfuwakai Asabu Heart and Cardiac Rehabilitation Clinic, Sapporo, Hokkaido Japan; 4 Department of Cardiovascular Medicine, National Hospital Organization Hakodate Hospital, Hakodate, Hokkaido Japan; 5 Department of Clinical Laboratory, Gunma Prefectural Cardiovascular Center, Maebashi, Gunma Japan; 6 Department of Cardiology, Gunma Prefectural Cardiovascular Center, Maebashi, Gunma Japan; 7 Department of Internal Medicine, Sapporo Kiyota Hospital, Sapporo, Hokkaido Japan

**Keywords:** Dynamic lung hyperinflation, Air trapping, Tidal volume, Cardiopulmonary exercise testing, Respiratory function

## Abstract

**Background:**

Many patients with heart disease potentially have comorbid chronic obstructive pulmonary disease (COPD); however, there are not enough opportunities for screening, and the qualitative differentiation of shortness of breath (SOB) has not been well established. We investigated the detection rate of SOB based on a visual and qualitative dynamic lung hyperinflation (DLH) detection index during cardiopulmonary exercise testing (CPET) and assessed potential differences in respiratory function between groups.

**Methods:**

We recruited 534 patients with heart disease or patients who underwent simultaneous CPET and spirometry (369 males, 67.0 ± 12.9 years) to scrutinize physical functions. The difference between inspiratory and expiratory tidal volume was calculated (TV E-I) from the breath-by-breath data. Patients were grouped into convex (decreased TV E-I) and non-convex (unchanged or increased TV E-I) groups based on their TV E-I values after the start of exercise.

**Results:**

Among the recruited patients, 129 (24.2%) were categorized in the convex group. There was no difference in clinical characteristics between the two groups. The Borg scale scores at the end of the CPET showed no difference. VE/VCO_2_ slope, its Y-intercept, and minimum VE/VCO_2_ showed no significant difference between the groups. In the convex group, FEV1.0/FVC was significantly lower compared to that in the non-convex group (69.4 ± 13.1 vs. 75.0 ± 9.0%). Moreover, significant correlations were observed between FEV1.0/FVC and Y-intercept (*r*=-0.343), as well as between the difference between minimum VE/VCO_2_ and VE/VCO_2_ slope (*r*=-0.478).

**Conclusions:**

The convex group showed decreased respiratory function, suggesting a potential airway obstruction during exercise. A combined assessment of the TV E-I and Y-intercept of the VE/VCO_2_ slope or the difference between the minimum VE/VCO_2_ and VE/VCO_2_ slopes could potentially detect COPD or airway obstruction.

## Background

Patients with cardiovascular diseases usually suffer from respiratory diseases such as chronic obstructive pulmonary disease (COPD) [1–3], and they develop fatigue and shortness of breath because of a variety of factors that limit exercise and activity, including lifestyle factors such as inactivity [4], increased left ventricular filling pressures [5], ventilatory-perfusion mismatch [6], and impaired oxygen delivery capacity due to cardiac dysfunction [7]. Current smoking or ex-smoking are common coronary risk factors and exacerbating factors of chronic heart failure [8–12]. In addition, poor lung function is an independent risk factor for cardiovascular disease and atrial fibrillation (AF) [13–17], and subclinical respiratory impairment is associated with the development of hypertension, which is a major risk factor for cardiovascular disease and mortality [18]. However, comorbid COPD without a history of smoking [19], potentially comorbid COPD, or respiratory impairment may not be adequately assessed, and this has not led to early detection of COPD or respiratory impairment in patients with heart disease [20].

Airflow obstruction in COPD is caused by decreased pulmonary elastic contractile pressure because of peripheral airway involvement and emphysematous lesions, resulting in collapsed airways and air trapped in the lungs during forced expiration (referred to as “air trapping”). Collapsed peripheral airways also occur during resting breathing as the disease progresses, contributing to lung hyperinflation [21]. In addition, air trapping, caused by collapsed peripheral airways, is strengthened with exertion or exercise, causing further lung hyperinflation. This is called dynamic lung hyperinflation (DLH) and is an important factor in patients with COPD, contributing to increased respiratory workload, shortness of breath on exertion, and reduced exercise tolerance [22, 23]. Although measurement of inspiratory capacity (IC) during exercise testing or the hyperventilation method has been proposed to assess DLH [24–29], opportunities for screening are insufficient. Nevertheless, there have been several reports combining exercise testing and inspiratory reserve capacity; however, there are limited reports on cardiopulmonary exercise testing (CPET), which is the most standard method of assessing exercise tolerance [30–35]. Furthermore, CPET, the gold standard for assessing exercise tolerance, provides information on the index of exercise tolerance. In CPET, the Y-intercept of the linear carbon dioxide production (V̇CO_2_) and minute ventilation (V̇E) relationship (the V̇E/V̇CO_2_ slope) is related to the severity of COPD and the forced expiratory volume (FEV) in 1 s (FEV1.0) as a percentage of forced vital capacity (FEV1.0/FVC) [36]. In our previous study, we have attempted to use CPET measurements to visually and qualitatively detect the DLH [37]. In this method, the difference between the expiratory and inspiratory tidal volumes is measured during incremental exercise, and expiration is assumed to be reduced relative to inspiration when DLH occurs.

In this study, we hypothesized that the presence or absence of the DLH index would be correlated with the CPET indices and respiratory function. We believe that by elucidating the relationship between the presence or absence of DLH indicators, CPET, and respiratory function, a comprehensive assessment of respiratory function using CPET is achievable. In addition, identifying respiratory impairment by CPET in asymptomatic or very mild stages of COPD will permit early preventive intervention and help prevent disease exacerbation.

## Methods

We included 534 patients with stable heart disease or patients who underwent simultaneous CPET and spirometry testing (age: 67.0 ± 12.9 years [95% confidence interval (CI): 65.9–68.1], height:161.9 ± 9.2 cm [95% CI: 161.2–162.7], body weight:62.5 ± 14.6 kg [95% CI: 61.3–63.7], Body mass index (BMI): 23.7 ± 4.4 kg/m^2^ [95% CI: 23.3–24.1]) to scrutinize physical functions.

The CPET was conducted following guideline-based methods [38], using a stationary bicycle (StrengthErgo 8; Mitsubishi Electric Engineering, Tokyo, COMBI 75XL3; Konami Sports Co., Ltd., Tokyo) and a breath-by-breath analysis with a gas analyzer (AE-300 S or AE-310 S; Minato Medical Science Co., Ltd., Tokyo). The maximal symptomatic exercise was performed using the ramp protocol. The exercise protocol consisted of 2–3 min of rest and 2–3 min of warm-up. The ramp protocol was adjusted to 10–20 W/min, assuming the individual exercise tolerance level. The rating of perceived exertion (RPE) at the end of the exercise was assessed using the Borg scale.

Furthermore, a breath-by-breath gas analyzer (AE-300 S or AE-310 S; Minato Medical Science Co., Ltd., Tokyo) was used to measure the ventilatory volume of each breath using a hot-wire flowmeter [39]. Before each exercise testing, we calibrated it according to standard protocols.

### Calculation of cardiopulmonary exercise testing measurements

We calculated the difference between inspiratory and expiratory tidal volumes (TV I and E, respectively) for each breathing from breath-by-breath data in each CPET [37]. Further, we have defined the difference between the expiratory and inspiratory tidal volumes as “TV E-I.” We plotted TV E-I against the time axis. In addition, we calculated the mean and standard deviation (S.D.) of TV E-I per minute based on the start of the warm-up (zero). Patients were categorized into two groups based on the TV E-I value: the convex group showing decreased TV E-I after the start of exercise, and the non-convex group displayed unchanged or increased TV E-I after the start of exercise.

We extracted other CPET parameters, such as the V̇E/V̇CO_2_ slope and its Y-intercept, minimum V̇E/V̇CO_2_, V̇O_2_/heart rate (HR), and dead-space gas volume to tidal volume ratio (VD/VT), for each case.

Respiratory compensation point (RCP) was comprehensively determined from the point where end-tidal carbon dioxide (ETCO_2_) decreases, V̇E/V̇CO_2_ begins to increase, and the inflection point of V̇E/V̇CO_2_ slope occurs [40, 41]. The relationship between V̇E and V̇CO_2_ was used to calculate the slope of linear regression (V̇E = aV̇CO_2_ + b), where “a” is the value of the V̇E vs. V̇CO_2_ slope, and “b” is the intercept on the V̇E axis (Y-intercept) [41, 42]. Minimum V̇E/V̇CO_2_ was determined as the nadir of the V̇E/V̇CO_2_ ratio during incremental exercise testing.

V̇O_2_/HR was calculated as V̇O_2_ divided by HR. VD/VT was calculated using the formula (ETCO_2_-FECO_2_)/ETCO_2_, incorporating end-tidal carbon dioxide (ETCO_2_) and mean expired carbon dioxide fraction (FECO_2_). Both indices were selected by taking the average of the resting, warm-up, and last minute of exercise.

### Spirometry testing

Spirometry testing was conducted following guideline-based standard methods [43], using respiratory function testing equipment (mainly electronic diagnostic spirometer Spiroshift SP-770 COPD Fukuda Denshi Co., Tokyo).

### Statistical analysis

Data were presented as mean ± standard deviation (S.D.) and 95% CIs. In addition, unpaired data were analyzed using the Student’s t-test. Moreover, paired data were analyzed using a paired t-test. Furthermore, plots of FEV1.0/FVC and Y-intercept of V̇E/V̇CO_2_ slope, the difference between minimum V̇E/V̇CO_2_ and V̇E/V̇CO_2_ slope were linearly regressed, and regression equations and coefficients were calculated. Statistical analyses were performed using Statistics for Excel 2012 (Social Survey Research Information Co., Tokyo, Japan).

### Ethics approval and consent to participate

The study was conducted following the principles outlined in the Declaration of Helsinki and approved by the ethical committees of Hakodate National Hospital (approval number: R4-0314001) and Gunma Prefectural Cardiovascular Center (approval number:2,022,020).

The need for informed consent was waived by the ethics committee of Hakodate National Hospital (approval number: R4-0314001) and Gunma Prefectural Cardiovascular Center (approval number:2,022,020) because of the retrospective nature of the study. The data obtained were delinked and anonymized, and this study was conducted using the data for analysis, with due consideration for protecting the participants’ personal information. The authors confirm that none of the participants could be identified and that they were fully anonymized. Furthermore, the authors affirm that all mandatory health and safety procedures complied with the course of conducting the experimental work reported in this paper.

## Results

There were no differences in physical parameters between the convex and non-convex groups; however, there were significantly more males in the convex group. Although there was no significant difference in smoking history between the two groups [Smoking history (+:-) Total 339:195, convex 85:44, non-convex 254:151, *p* = 0.514], the convex group had more severe cases when the GOLD classification was applied [GOLD classification (0:I: II: III: IV); total 397:86:39:9:3, convex 79:24:15:8:3, non-convex 318:62:24:1:0, *p* < 0.001; Table 1].

**Table 1:** Data of participants’ clinical characteristics

		Total *n* = 534			Convex *n* = 129			Non-convex *n* = 405		p value
		Mean ± S.D	95% CI		Mean ± S.D	95% CI		Mean ± S.D	95% CI	
Sex	M: F	369:165			104:25			265:140		0.002
Age	[years]	67.0 ± 12.9	[65.9–68.1]		68.2 ± 11.7	[66.2–70.3]		66.6 ± 13.3	[65.3–67.9]	0.215
Height	[cm]	161.9 ± 9.2	[161.2-162.7]		163.1 ± 9.0	[161.6-164.7]		161.5 ± 9.2	[160.7-162.4]	0.087
Body weight	[kg]	62.5 ± 14.6	[61.3–63.7]		64.6 ± 15.3	[62-67.2]		61.8 ± 14.3	[60.4–63.2]	0.059
BMI		23.7 ± 4.4	[23.3–24.1]		24.2 ± 4.6	[23.4–24.9]		23.5 ± 4.3	[23.1–24.0]	0.169
NYHA classification	[0:I: II: III: IV]	97:213:155:69:0			20:48:46:15:0			77:165:109:54:0		0.288
Ischemic heart disease		165 (30.9)			42 (32.6)			123 (30.4)		0.662
Valve disease		56 (10.5)			10 (7.8)			46 (11.4)		0.322
Chronic heart failure		154 (28.8)			43 (33.3)			111 (27.4)		0.267
Atrial fibrillation		32 (6.0)			10 (7.8)			22 (5.4)		0.393
Others		52 (9.7)			14 (10.9)			38 (9.4)		0.612
Normal participants		97 (18.2)			20 (15.5)			77 (19.0)		0.432
LVEF	[%]	55.8 ± 14.4	[54.6–57.0]		55.8 ± 13.5	[53.4–58.2]		55.8 ± 14.7	[54.3–57.2]	0.927
BNP	[pg/dL]	150.4 ± 247.6	[126.8–174.0]		136.1 ± 162.4	[104.9-167.3]		154.2 ± 267.9	[125.1-183.2]	0.550
Smoking history		339 (63.5)			85 (65.9)			254 (62.7)		0.531
bronchial inhalers		75 (14.0)			21 (16.3)			54 (13.3)		
GOLD classification	[0:I: II: III: IV]	397:86:39:9:3			79:24:15:8:3			318:62:24:1:0		< 0.001

Data are presented as mean ± S.D. and 95% CI. NYHA classification of Normal participants was counted as “0”. There was no difference in NYHA classification between the two groups. The convex group had more males and significantly more severe cases according to the GOLD classification. No significant differences in other clinical characteristics were observed between the two groups

CI: confidence interval, BMI: body mass index, NYHA: New York Heart Association, LVEF, Left ventricular ejection fraction, BNP: brain natriuretic peptide, GOLD: Global Initiative for Chronic Obstructive Lung Disease

Furthermore, all the participants underwent symptomatic maximal CPET. Moreover, there were 129 patients in the convex group with decreased TV E-I during CPET and 405 patients in the non-convex group. Although the RPE at the end of the exercise test showed no difference in shortness of breath between the two groups, lower extremity fatigue was significantly lower in the convex group.

### The indices of cardiopulmonary exercise testing

A list of typical CPET parameters is shown in Table 2. There were no significant differences in the exercise tolerance indices between the two groups. Although there were no differences in the V̇E/V̇CO_2_ slope and Y-intercept between the two groups, the minimum V̇E/V̇CO_2_ tended to be higher in the convex group.

**Table 2:** Cardiopulmonary exercise testing indices

		Total *n* = 534			Convex *n* = 129			Non-convex *n* = 405		p value
		Mean ± S.D	95% CI		Mean ± S.D	95% CI		Mean ± S.D	95% CI	
RPE– leg		17.2 ± 2.3	[17.0-17.4]		16.7 ± 2.4	[16.3–17.1]		17.3 ± 2.3	[17.1–17.5]	0.011
RPE - chest		15.9 ± 2.6	[15.7–16.1]		16.0 ± 2.5	[15.5–16.4]		15.9 ± 2.7	[15.6–16.2]	0.755
Peak V̇O_2_	[ml/min]	1057 ± 536	[1011–1102]		1122 ± 764	[990–1254]		1036 ± 439	[993–1079]	0.112
Peak V̇O_2_/W	[ml/kg/min]	16.8 ± 7.2	[16.2–17.5]							
%Peak V̇O_2_	[%]	71.5 ± 23.9	[69.5–73.6]		73.3 ± 32.8	[67.6–78.9]		71.0 ± 20.2	[69.0–73.0]	0.352
Peak HR	[bpm]	125.1 ± 25.5	[123.0-127.3]		125.9 ± 23.7	[121.8–130.0]		124.9 ± 26.1	[122.3-127.4]	0.698
Peak RER		1.15 ± 0.1	[1.14–1.16]		1.13 ± 0.09	[1.11–1.14]		1.16 ± 0.1	[1.15–1.17]	0.001
ΔV̇O_2_/ΔWR		8.8 ± 1.9	[8.6-9.0]		8.7 ± 1.9	[8.4-9.0]		8.8 ± 1.9	[8.6-9.0]	0.540
V̇E/V̇CO_2_ slope		35.5 ± 9.5	[34.7–36.3]		35.1 ± 8.8	[33.6–36.7]		35.2 ± 7.5	[34.5–36.0]	0.923
Y-intercept		4.3 ± 3.3	[4.0-4.6]		4.6 ± 3.0	[4.1–5.1]		4.4 ± 2.9	[4.1–4.6]	0.423
minimum V̇E/V̇CO_2_		38.6 ± 9.1	[36.5–40.6]		42.0 ± 10.6	[37.4–46.7]		37.5 ± 8.3	[35.3–39.7]	0.056
rest RR		17.0 ± 4.4	[16.6–17.4]		17.6 ± 3.5	[17-18.2]		16.8 ± 4.6	[16.3–17.2]	0.070
peak RR		34.5 ± 8.7	[33.8–35.3]		33.3 ± 7.4	[32.1–34.6]		34.9 ± 9.1	[34.0-35.8]	0.074
rest VD/VT		0.42 ± 0.06	[0.41–0.42]		0.43 ± 0.06	[0.42–0.44]		0.41 ± 0.06	[0.41–0.42]	0.061
peak VD/VT		0.34 ± 0.05	[0.34–0.35]		0.34 ± 0.05	[0.33–0.35]		0.34 ± 0.05	[0.34–0.35]	0.793
rest V̇O_2_/HR		2.9 ± 0.8	[2.9-3.0]		3.0 ± 0.7	[2.9–3.1]		2.9 ± 0.8	[2.8-3.0]	0.391
Peak V̇O_2_/HR		8.1 ± 2.8	[7.8–8.3]		8.3 ± 2.7	[7.8–8.8]		8.0 ± 2.8	[7.7–8.3]	0.348

Data are presented as mean ± S.D. and 95% CI. The rating of perceived exertion at the end of the exercise test showed no difference in shortness of breath between the two groups. In contrast, lower extremity fatigue was significantly lower in the convex group. Regarding the cardiopulmonary exercise test indices, there were almost no differences between the two groups; however, the minimum V̇E/V̇CO_2_ ratio tended to be higher in the convex group

CI, confidence interval; RPE, rate of perceived exertion; V̇O_2_, oxygen uptake; Peak V̇O_2_/W, peak oxygen uptake per weight; V̇CO_2_, carbon dioxide; HR, heart rate; RER, respiratory exchange ratio; WR, work rate; RR, respiratory rate; VD/VT, deadspace gas volume to tidal volume ratio

### The indices of spirometry testing

A list of typical spirometry testing parameters is shown in Table 3. Vital capacity (VC), tidal volume (TV), expiratory reverse volume (ERV), and inspiratory reverse volume (IRV) did not differ significantly. However, the convex group had significantly lower FEV1.0/FVC and predictive rates for FEV1.0/FVC, % PEF, and % MVV.

**Table 3:** Spirometry testing indices

		Total *n* = 534			Convex *n* = 129			Non-convex *n* = 405		p value
		Mean ± S.D	95% CI		Mean ± S.D	95% CI		Mean ± S.D	95% CI	
VC	[L]	3.12 ± 0.84	[3.05–3.19]		3.18 ± 0.81	[3.04–3.32]		3.10 ± 0.86	[3.01–3.18]	0.363
%VC	[%]	103.2 ± 18.7	[101.6-104.8]		104.6 ± 18.6	[101.3-107.8]		105.6 ± 18.7	[103.8-107.4]	0.573
TV	[L]	0.91 ± 0.37	[0.87–0.94]		0.94 ± 0.45	[0.86–1.02]		0.90 ± 0.34	[0.86–0.93]	0.246
ERV	[L]	0.96 ± 0.48	[0.92-1.00]		0.95 ± 0.44	[0.88–1.03]		0.96 ± 0.49	[0.91–1.01]	0.872
%ERV	[%]	76.5 ± 34.0	[73.6–79.3]		72.6 ± 31.3	[67.2–78.0]		77.7 ± 34.8	[74.3–81.1]	0.142
IRV	[L]	1.25 ± 0.58	[1.20–1.30]		1.28 ± 0.54	[1.19–1.37]		1.24 ± 0.59	[1.18–1.30]	0.511
IC	[L]	2.16 ± 0.64	[2.10–2.21]		2.22 ± 0.63	[2.11–2.33]		2.14 ± 0.64	[2.07–2.20]	0.207
FVC	[L]	3.06 ± 0.85	[2.98–3.13]		3.11 ± 0.83	[2.97–3.25]		3.04 ± 0.85	[2.95–3.12]	0.395
%FVC	[%]	92.4 ± 16.6	[91.0-93.8]		91.4 ± 17.3	[88.5–94.4]		92.7 ± 16.3	[91.1–94.3]	0.451
FEV1.0	[L]	2.26 ± 0.72	[2.20–2.32]		2.17 ± 0.74	[2.05–2.30]		2.28 ± 0.71	[2.21–2.35]	0.130
%FEV1.0	[%]	101.5 ± 23.4	[99.5-103.4]		96.2 ± 28.0	[91.4–101.0]		103.1 ± 21.5	[101.0-105.2]	0.003
FEV1.0/FVC	[%]	73.7 ± 10.4	[72.8–74.6]		69.4 ± 13.1	[67.1–71.6]		75.0 ± 9.0	[74.2–75.9]	0.000
MMF	[L/s]	2.02 ± 1.06	[1.78–2.27]		1.64 ± 0.83	[1.26–2.03]		2.10 ± 1.06	[1.82–2.38]	0.100
%MMF	[%]	68.7 ± 28.5	[62.2–75.2]		58.3 ± 28.1	[45.3–71.3]		70.3 ± 25.1	[63.7–77.0]	0.091
PEF	[L/s]	6.33 ± 2.34	[5.80–6.86]		5.54 ± 2.15	[4.60–6.48]		6.61 ± 2.36	[5.99–7.24]	0.079
%PEF	[%]	83.6 ± 23.1	[78.4–88.8]		72.8 ± 24.8	[61.9–83.7]		87.5 ± 21.4	[81.9–93.2]	0.014
MVV	[L/min]	68.6 ± 25.7	[66.1–71.0]		74.8 ± 30.3	[69-80.6]		81.0 ± 28.3	[77.9–84.0]	0.058
%MVV	[%]	84.7 ± 24.7	[82.4–87.0]		92.2 ± 31.2	[86.2–98.2]		103.2 ± 27.1	[100.3-106.1]	0.001

Data are presented as mean ± S.D. and 95% CI. The measured values of forced expiratory volume in 1 s as a percentage of forced vital capacity (FEV1.0/FVC), predicted values of forced expiratory volume in 1 s (%FEV1.0), peak expiratory flow (%PEF), and maximal ventilatory volume (%MVV) were significantly lower in the convex group

CI, confidence interval; VC, vital capacity; VT, tidal volume; ERV, expiratory reserve volume; IRV, inspiratory reserve volume; IC, inspiratory capacity; FVC, forced vital capacity; FEV, forced expiratory volume; FEV1.0, forced expiratory volume in 1 s; FEV1.0/FVC, forced expiratory volume in 1 s as a percentage of forced vital capacity; MMF, maximal mid-expiratory flow; PEF, peak expiratory flow; MVV, maximal ventilatory volume

### Relationship between exhaled gas analysis index and spirometry testing

**Fig. 1:**
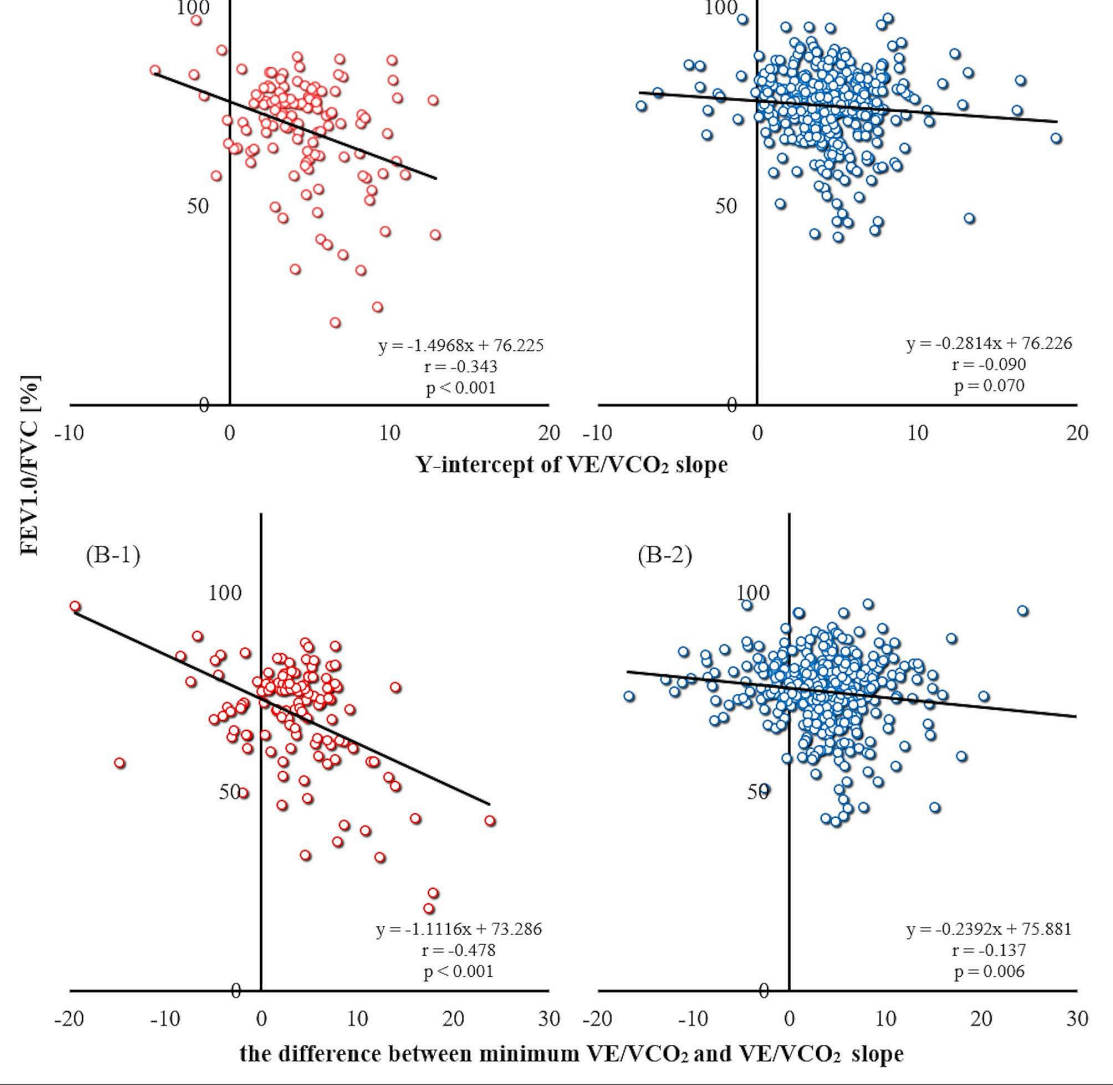
Correlation between respiratory function and cardiopulmonary exercise testing (CPET) indices. The upper two panels show the relationship between FEV1.0/FVC and the Y-intercept of V̇E/V̇CO_2_ in the convex group (**A**-**1**) and the non-convex group (**A**-**2**). The lower two panels show the relationship between FEV1.0/FVC and the difference between the minimum V̇E/V̇CO_2_ and V̇E/V̇CO_2_ slopes in the convex (**B**-**1**) and non-convex groups (**B**-**2**). V̇E/V̇CO_2_: ventilatory equivalent for carbon dioxide production; FEV1.0/FVC: forced expiratory volume in 1 s as a percent of forced vital capacity;

In the convex group, Y-intercept and FEV1.0/FVC showed a significant negative correlation (convex; *r*=-0.343 [-0.487 ≦ ρ≦-0.181], *p* < 0.001, non-convex; *r*=-0.090 [-0.186 ≦ ρ ≦ 0.008], *p* = 0.070; Fig. 1 (A-1, 2)).

V̇E/V̇CO_2_ slope and minimum V̇E/V̇CO_2_ showed insignificant correlation with FEV1.0/FVC; however, there was a significant negative correlation with the difference between V̇E/V̇CO_2_ slope and minimum V̇E/V̇CO_2_ (convex: *r*=-0.478 [-0.601 ≦ ρ≦-0.333], *p* < 0.001; non-convex: *r*=-0.137 [-0.231 ≦ ρ≦-0.040], *p* = 0.006; Fig. 1 (B-1, 2)).

## Discussion

This study stands out as one of the few studies examining the relationship between differences in respiratory function and CPET indices, based on the qualitative detection of DLH using CPET. DLH is a physiological respiratory mechanism in which expiration occurs less than inspiration, with an increased respiratory rate. Past studies have measured a decrease in IC or an increase in EELV during exercise [44]. Nevertheless, our study reveals that DLH can be readily detected and correlated with respiratory function by analyzing data from exhaled gas analyses, thereby supporting the underlying theory.

### Respiratory function by spirometry testing

Respiratory functions, such as VC and TV, did not differ between the two groups; however, FEV1.0/FVC, % MVV, and % PEF were lower in the convex group. In the convex group, the mean value of FEV1.0/FVC was equivalent to the diagnostic criteria for obstructive ventilatory impairment. In contrast, in the non-convex group, most cases did not meet the criteria for GOLD stage 1, and several cases were not diagnosed as having obstructive ventilation impairment on spirometry testing. Nevertheless, several patients in the convex group likely had DLH because of peripheral airway obstruction (stenosis) or other factors, as they had less expiration than inspiration during the exercise. Therefore, it is possible that respiratory function had already declined before meeting the criteria for diagnosis of DLH, due to the obstruction of the small bronchioles and other organs. Furthermore, it has already been shown that subclinical respiratory impairment may also affect cardiovascular function and cardiovascular disease [13–18]. Although this was a cross-sectional study and the outcome of very mild cases was unknown, we believe that TV E-I may be a qualitative indicator of peripheral airway obstruction.

### Relationship between cardiopulmonary exercise testing indices and dynamic pulmonary hyperinflation

Ventilatory efficiency decreases because of congestion caused by heart failure and obstructive ventilation impairment [41]. For instance, in patients with heart failure, the minimum V̇E/V̇CO_2_ and V̇E/V̇CO_2_ slope increase with disease severity; however, they are generally consistent [45]. In contrast, in COPD, the V̇E/V̇CO_2_ slope increases in mild disease but decreases in severe disease [46, 47]. Furthermore, in COPD, the Y-intercept of the V̇E/V̇CO_2_ slope is related to FEV1.0/FVC and the Y-intercept is higher [36]. Murata et al. reported that as COPD progresses, the minimum V̇E/V̇CO_2_ and V̇E/V̇CO_2_ slopes may diverge, or the V̇E/V̇CO_2_ slope may become pseudo-negative, and the Y-intercept may be high [36, 48]. However, the decrease in IC or increase in EELV during exercise is fundamental to the evaluation of DLH; both indices only report observational studies on COPD and do not examine the presence or absence of DLH or its extent.

Although the convex group showed a trend towards a higher minimum V̇E/V̇CO_2_ in this study, there were no significant differences in these indices between the two groups, including the Y-intercept and V̇E/V̇CO_2_ slope. However, even in such cases, FEV1.0/FVC and the Y-intercept of the V̇E/V̇CO_2_ slope showed a significant correlation in the convex group, similar to the results of previous studies. Furthermore, FEV1.0/FVC also showed a significant negative correlation with the difference between the minimum V̇E/V̇CO_2_ ratio and the V̇E/V̇CO_2_ slope. In contrast, in the non-convex group, the correlation between the difference in minimum V̇E/V̇CO_2_ and V̇E/V̇CO_2_ slope and FEV1.0/FVC was very limited, indicating that a combined evaluation with TV E-I is crucial.

Although the study group had milder respiratory function impairment than those in previous studies, it was suggested that the combined TV E-I and Y-intercept or the difference between the minimum V̇E/V̇CO_2_ and V̇E/V̇CO_2_ slope during incremental exercise testing could provide an index of respiratory function and an assessment of the severity of peripheral airway obstruction in patients with stable cardiac disease.

### The usefulness of detecting dynamic lung hyperinflation using cardiopulmonary exercise test

Most participants in this study had stable heart disease and mild respiratory function impairment. However, 63.4% of the patients had a smoking history, and more than 20% showed the possibility of DLH on CPET. Interestingly, the prevalence of COPD is expected to decrease in high-income countries as smoking declines; however, it will become a major social problem in low-to-middle-income countries [49]. COPD causes chronic systemic inflammation, leading to a decline in physical function and a worsening prognosis [50]. Moreover, DLH increases the respiratory workload and restricts venous return [51], leading to exercise limitation and static lung hyperinflation caused by COPD progression and a worsened prognosis [49, 52, 53].

We believe that capturing respiratory changes associated with increased exercise intensity using CPET, as demonstrated in this study, provides a simple and qualitative method for detecting airway stenosis and DLH at an early stage, even in patients with mild symptoms or asymptomatic disease. Furthermore, we believe this will lead to early scrutiny, appropriate therapeutic interventions, and drug prescriptions, ultimately improving the quality of life and patient prognosis.

### Limitations

There are some limitations to this study. First, this was a cross-sectional study; moreover, cardiopulmonary exercise and spirometry testing were not often performed at approximately the same time, possibly resulting in a selection bias (comorbidity of DLH). In addition, the course of the patients’ conditions in this study was unclear, including whether there was a worsening shortness of breath and other symptoms, a progressive decline in respiratory function, or a diagnosis of COPD. Therefore, further studies are warranted.

Second, the study did not compare the results with those of the existing DLH assessment methods or evaluate the response to bronchodilator use. Therefore, it is difficult to confirm the presence of DLH based on the results of this study alone.

Third, several participants in this study had relatively preserved respiratory function. Therefore, it is uncertain whether a similar trend would be observed in patients with moderate-to-severe COPD who have already been diagnosed. Finally, it is currently difficult to determine the severity of DLH; therefore, developing appropriate analytical methods for TV E-I is desirable.

## Conclusion

Evaluating data on differences in TV E-I during cardiopulmonary exercise testing has proven useful for DLH in patients with stable heart disease. The combined evaluation of the TV E-I and Y-intercept of the V̇E/V̇CO_2_ slope or the difference between the minimum V̇E/V̇CO_2_ and V̇E/V̇CO_2_ slopes in CPET, could detect cases of potential respiratory impairment or peripheral airway obstruction.

## Data Availability

The dataset used in this study is available from the corresponding author upon request.

## Abbreviations

VAT: ventilatory anaerobic threshold
Inc-Ex: incremental exercise
CPET: Cardiopulmonary exercise testing
V̇O_2_: Oxygen uptake
V̇CO_2_: carbon dioxide production
V̇E: ventilatory equivalent
RPE: Rating of perceived exertion
RR: respiratory rate
VD/VT: dead-space gas volume to tidal volume ratio
COPD: chronic obstructive pulmonary disease
DLH: dynamic lung hyperinflation
TV: tidal volume
TV I: inspiratory tidal volume
TV E: expiratory tidal volume
FEV: forced expiratory volume
FEV1.0: forced expiratory volume in 1 s
FEV1.0/FVC: forced expiratory volume in 1 s as a percent of forced vital capacity
VC: vital capacity
IC: inspiratory capacity
S.D.: standard deviation
CI: confidence interval
